# Next-Generation Sequencing—Optimal Sequencing of Therapies in Relapsed/Refractory Chronic Lymphocytic Leukemia (CLL)

**DOI:** 10.1007/s11912-023-01454-w

**Published:** 2023-09-08

**Authors:** Florian Simon, Jan-Paul Bohn

**Affiliations:** 1 Department I of Internal Medicine and Center of Integrated Oncology Aachen, Bonn, Cologne, Düsseldorf, German CLL Study Group, Faculty of Medicine and University Hospital of Cologne, University of Cologne, Cologne, Germany; 2 Department of Internal Medicine V, Hematology and Oncology, Comprehensive Cancer Center Innsbruck (CCCI), Medical University of Innsbruck, Anichstrasse 35, A-6020 Innsbruck Innsbruck, Austria

**Keywords:** Relapsed/refractory disease, Therapy sequence, Venetoclax, BTK inhibitor, Acalabrutinib, Zanubrutinib, Pirtobrutinib, Nemtabrutinib

## Abstract

**Purpose of Review:**

This research paper aims to provide an overview of evidence-based sequencing of therapies in relapsed/refractory chronic lymphocytic leukemia (CLL) in the era of targeted drugs.

**Recent Findings:**

In the absence of data from randomized clinical trials comparing novel agents head-to-head, growing evidence suggests that patients with late relapse (> 2 years) after fixed-duration therapies benefit from identical retreatment, whereas a class switch is favorable in those with short-lived remissions or progressive disease on continuous drug intake. Treatment of patients previously exposed to both covalent inhibitors of BTK and BCL2 remains an unmet medical need. Novel drugs, in particular noncovalent BTKI, show promising efficacy in this difficult-to-treat subgroup in early clinical trials.

**Summary:**

The optimal sequencing of therapies in CLL requires consideration of individual patient factors and disease characteristics. Double-refractory disease continuous to pose a clinical challenge with a focus on participation in clinical trials whenever possible.

## Introduction

Tremendous progress has been made with the implementation of targeted agents in the treatment landscape of chronic lymphocytic leukemia (CLL) in recent years. Inferior in terms of both efficacy and tolerability, conventional chemoimmunotherapy (CIT) is no longer recommended in first and subsequent lines of therapy in most patients [1–4]. Foremost, covalent inhibitors of Bruton’s tyrosine kinase (BTKI) and anti-apoptotic protein B-cell lymphoma 2 (BCL2) shifted in the forefront of widely accepted treatment algorithms [5]. Notably, CLL patients with high-risk genetic alterations such as unmutated immunoglobulin heavy chain variant region (IGHV) status, TP53 aberrations and/or a complex karyotype derive major benefit with these novel agents compared to CIT [6, 7•]. Nevertheless, clonal evolution giving rise to acquired resistance and drug-associated toxicity remain major concerns in daily clinical practice commonly exposing CLL patients to multiple lines of therapy in the course of their disease. In particular, genetic high-risk disease features retain their adverse prognostic impact with currently licensed novel agents as shown by inferior survival outcomes in affected CLL patients in the long run [8–10]. According to the National Comprehensive Cancer Network (NCCN) and the European Society of Medical Oncology (ESMO) guidelines, treatment options for relapsed/refractory (r/r) CLL are nearly identical to those used in untreated disease. Thus, treatment recommendations for second and subsequent lines of therapy majorly depend on previous drug exposure and tolerability. In this review, we discuss therapeutic options available in r/r CLL, including investigational drugs, and provide a framework to help guide sequencing of drugs in individual patients.

## Sequencing Treatment Strategies in Relapsed/Refractory CLL in 2023

Before choosing subsequent treatment for any patient with r/r CLL, it remains critical to assess whether treatment indication according to iwCLL 2018 criteria is fulfilled [11]. Patients with asymptomatic disease and no significant anemia (< 100 g/l hemoglobin) or thrombocytopenia (< 100x10^9^/l platelets) do not benefit from timely treatment initiation and may rather be monitored for further disease progression. As soon as the patient meets iwCLL2018 criteria to initiate therapy, re-assessment of the biological risk profile, particularly TP53 aberrations in terms of TP53 mutational status if previously wild-type and deletion 17p via karyotype/FISH, is essential to help guide therapy selection. CIT frequently allows for selection of TP53 aberrant subclones and emergence of karyotype complexity diminishing susceptibility to CIT re-exposure [12]. Notably, broad availability of targeted agents with clearly superior efficacy in genetic high-risk disease outperformed CIT-based retreatments in almost all patients naive to these novel drugs [8, 13].

## Selecting CLL Treatment After Previous CIT in Patients Naive to Novel Drugs

Long-term follow-up data for efficacy and toxicity exist for both BTKI and venetoclax-based treatments in the r/r setting after CIT [13, 14, 15•, 16••]. Given the increased risk for severe immune-mediated toxicity and infections, currently licensed PI3K inhibitors are generally reserved for patients intolerant or refractory to these agents [17, 18].

Ibrutinib was the first covalent BTKI licensed for patients with r/r CLL back in 2014. In the recent final analysis of the confirmatory randomized phase 3 RESONATE trial comparing continuous ibrutinib with the CD20-antibody ofatumumab given for 24 weeks demonstrated superior overall response rate (ORR), progression-free survival (PFS) and overall survival (OS) with a median follow-up of 65.3 months. With a median time on ibrutinib treatment of 41 months, the prevalence of most grade ≥3 adverse events (AEs) of clinical interest such as cytopenias, infections, atrial fibrillation and diarrhea generally declined in years 1 to 6 from study entry. The prevalence of grade ≥3 hypertension and major hemorrhage, however, remained an ongoing issue throughout the study (4 to 11% and 2 to 3% from year 1 to 6, respectively). Overall, 16% of patients discontinued ibrutinib due to AEs and the frequency of drug discontinuation remained stable over time with 6 to 4% of patients from year 1 to 6 after study entry [14]. In the subgroup of patients with deletion17p, long-term follow-up of the phase 2 RESONATE-17 study in 144 patients reported an ORR of 83% and a 2-year PFS of 63% illustrating the prevailing effect of ibrutinib in this high-risk subpopulation [7•].

The second-generation covalent BTKI acalabrutinib was approved for patients with r/r CLL in 2019 based on the randomized phase 3 ASCEND study comparing acalabrutinib with investigator’s choice of bendamustin-rituximab (BR) or idelalisib-rituximab (IR). In the final analysis and a median follow-up of 41 months ORR including partial response (PR) with lymphocytosis was 92% with acalabrutinib transforming in a 42-month PFS of 62%. Still, patients with deletion 17p succumb to a shorter median PFS of 36 months in contrast to not reached for the complete study cohort [15•]. The first randomized phase 3 head-to-head comparison with ibrutinib (ELEVATE-RR) suggested similar efficacy but superior tolerability with acalabrutinib. Notably, acalabrutinib was associated with lower diarrhea (35% versus 46%), hypertension (9% versus 23%), arthralgia (16% versus 23%), atrial fibrillation (9% versus 16%) and bleeding events (38% versus 51%). There were two cardiac arrests seen with ibrutinib, none with acalabrutinib. Finally, patients more frequently discontinued ibrutinib than acalabrutinib due to AEs (21% versus 16%) [19••].

At ASH 2022, the final analysis of the phase 3 ALPINE study directly randomizing the second-generation covalent BTKI zanubrutinib versus ibrutinib in r/r CLL patients was presented. ORR was significantly higher with zanubrutinib (86.2% versus 75.7%, *p*=0.0007) translating in a significantly prolonged PFS at two years (79.5% versus 67.3%, *p*=0.0024) with a median follow-up of 29.6 months. Notably, this benefit in PFS prevailed in the subgroup of patients with a TP53-alteration (77.6% versus 55.7%, *p*=0.134) and was stable across all subgroups in favor of zanubrutinib. Whereas grade 3 to 5 AEs seemed about even in both study arms (67.3% versus 70.4%) leading to dose interruption in 50.0% and 56.8% of cases, respectively, there was a trend towards fewer treatment discontinuations due to AEs with zanubrutinib (15.4% versus 22.2%). In terms of AEs of special interest, all grade atrial fibrillation was indeed less frequently seen with zanubrutinib (5.2% versus 13.3%) whereas there appeared to be no difference in the incidence of all grade hemorrhage (42.3% versus 41.4%) and arterial hypertension (23.5% versus 22.8%). All grade neutropenia appeared to be more frequent with zanubrutinib without increase of infections (29.3% versus 24.4% and 71.3% versus 73.1%, respectively). Finally, there were no fatal cardiac AEs seen with zanubrutinib in contrast to 6 fatal cardiac disorders on ibrutinib [16••].

Overall, the superior tolerability and at least comparable efficacy profile favor the use of second-generation covalent BTKI over ibrutinib in most patients, particularly in those with an increased cardiovascular risk profile.

In patients with prior bleeding complications, dual antiplatelet treatment, ventricular arrhythmias and/or advanced heart failure, however, BTKI treatment should generally be reconsidered, and venetoclax-based regimes be preferred.

Besides covalent BTKI, the BCL2-inhibitor venetoclax is the most appealing alternative treatment option for patients having failed CIT. After initial reports on impressive efficacy when given continuously as monotherapy with high ORR > 70% irrespective of the TP53 functional status as well as deep responses, including negativity for minimal residual disease (MRD, < 10^-4^ malignant cells), investigation of venetoclax has advanced to randomized clinical trials in combination with a CD20-antibody allowing a time-limited treatment approach [20, 21]. The current standard of 2 years venetoclax in combination with 6 months rituximab (VenR) was established in the randomized phase 3 MURANO study against 6 cycles of BR. At ICML 2023, the final analysis with 7 years follow-up was presented. Mayor benefits in median PFS (54.7 versus 17.0 months) and OS (7-year OS: 69.6% versus 51.0%) were sustained despite allowed crossover design. Median PFS in the subgroup of patients with TP53 alterations was 30 months. Although MRD negativity at the end of treatment translated in prolonged PFS with VenR (52.5% versus 29.3%, *p*<0.0001), there was no significant difference in OS further supporting VenR as licensed non-MRD-guided regimen [22••].

The use of PI3K inhibitors for r/r CLL patients has widely faded in the background given the concerning immune-mediated toxicity profile including diarrhea, pneumonitis, and transaminitis in the short term and a non-diminishing risk of colitis and pneumonitis with prolonged drug exposure [18, 23]. There are two PI3K delta inhibitors currently approved in this setting, idelalisib and duvelisib. However, despite the PFS advantages seen in the registration trials, a recent analysis from the US FDA Oncologist Drugs advisory Committee (ODAC) meeting suggested a trend towards inferior long-term OS compared with the CIT control arms, questioning their persistent use in CLL [24].

## Selecting CLL Treatment at Relapse After Novel Drugs

In r/r CLL patients previously exposed to novel agents, the first step is to identify whether treatment was discontinued due to toxicity or disease progression on- or off-drug depending on whether a continuous or time-limited regimen was administered.

## Re-treatment—Never Change a Winning Team?

In patients having discontinued previous indefinite covalent BTKI due to toxicity, it is essential to reconsider if the patient is actually in need for direct subsequent therapy according to iwCLL 2018 criteria. This is highlighted by longer follow-up of patients having discontinued frontline ibrutinib due to toxicity showing a median time to disease progression of 23 months [25]. When subsequent treatment is indicated in such patients, selecting an alternative covalent BTKI appears feasible. Acalabrutinib was prospectively evaluated in 33 patients with ibrutinib intolerance demonstrating encouraging tolerability with no treatment discontinuations due to AEs while prevailing a favorable efficacy with a 2-year PFS of 72%. Moreover, the 2-year survival rate among these patients was reported to be 81% [26•]. Similarly, a phase 2 study of zanubrutinib in 67 patients with B-cell malignancies after intolerance to ibrutinib and/or acalabrutinib demonstrated stable disease control or deepening remissions with no treatment discontinuations due to AEs while 70% of ibrutinib and 83% of acalabrutinib associated intolerance events did not recur with zanubrutinib [27•].

Before starting with an alternative covalent BTKI in intolerant patients at relapse, however, it is recommended to test for acquired resistance mutations in the BTK binding pocket and for gain-of-function mutations of PLCG2.These are found in 57–87% of patients progressing on ibrutinib and at a similar frequency on acalabrutinib, implying treatment resistance for the entire class of currently licensed covalent BTKI [28–30].

In CLL patients with progressive disease after fixed-duration venetoclax in combination with an anti-CD20-antibody and a duration of response beyond 2 years, retreatment appears effective and feasible. Recently presented at ICML 2023, 25 patients progressing after VenR were subsequently re-treated after a median treatment-free interval of 2.3 years in a substudy of the aforementioned phase 3 MURANO trial achieving an impressive ORR of 72% and a median PFS of 23.3 months [22••]. Supporting this trend, a retrospective study involving 46 patients (including 11 patients from the MURANO trial) confirmed an ORR of 79.5% to re-exposure and a median PFS of 25 months with a median follow-up of 10 months [31].

These findings suggest that re-exposure to fixed-duration venetoclax combined with an anti-CD20-antibody may be considered a viable therapeutic option for patients experiencing late relapse after initial treatment. Accordingly, the recently initiated ReVenG phase 2 trial (NCT04895436) as well as retreatment within the CLL2-BAG phase 2 trial (NCT02401503) aim to better elucidate the role of fixed-duration retreatment with venetoclax plus obinutuzumab in CLL.

## Venetoclax +/- Anti-CD20-antibodies After BTK-Inhibition

In CLL patients with disease progression on covalent BTKI treatment it is appropriate to switch the drug class to venetoclax-based regimens. However, it is critical to not prematurely discontinue BTKI until the target dose of venetoclax is reached to reduce the risk of potential disease flare or rapid disease progression associated with prompt BTKI stop [32].

This recommendation is based on a phase 2 study of 91 CLL patients who had relapsed on continuous ibrutinib and now received venetoclax monotherapy demonstrating a promising ORR of 65% and a median PFS of 24.7 months. Undetectable MRD was achieved in 42% of patients available for analysis at 24 weeks [33]. Further evidence for venetoclax monotherapy comes from real-world data in the UK with an ORR of 85% (CR 23%) and a 1-years PFS of 65% in 63 CLL patients progressing on BTKI treatment as well as the US with an ORR of 85% in 62 patients with short follow-up [34, 35].

Prospective data for a time-limited approach of venetoclax in combination with an anti-CD20-antibody in previously BTKI-exposed CLL patients is still lacking. However, in a multicenter analysis of the UK and the US among 321 heavily-pretreated CLL patients, including 79% with prior ibrutinib therapy, ORR (81% vs. 84%), estimated 12-month PFS and OS (hazard ratio 1.0 for PFS and 1.2 for OS) were similar for venetoclax monotherapy (*n*=270) and venetoclax combinations (*n*=51, 38 rituximab, 13 obinutuzumab), respectively [36].

In our practice, we currently favor start of combination treatment in most patients and consider continued venetoclax application in those not achieving undetectable MRD at the end of the planned treatment period.

## BTK-Inhibition After Venetoclax - Anti-CD20-antibodies

In CLL patients with early relapse (< 2 years) after fixed-duration treatment with venetoclax plus anti-20-antibody or refractory disease on venetoclax intake, a class-switch to covalent BTKI is recommended as mechanisms of acquired resistance seem hardly overlapping [37].

Although clinical evidence is limited, two retrospective analysis suggest efficacy and feasibility of this approach with an ORR of 91% and a median PFS of 34 months in 23 CLL patients pretreated with venetoclax and an ORR of 84% and a median PFS of 32 months in 44 CLL patients with a median of 3 previous therapies, including venetoclax. Of note, most of the patients included in these trials were treated with ibrutinib (*n*=21/23 and *n*=43/44) [38, 39].

## Double-Refractory CLL Previously Exposed to Both Inhibitors of BTK and BCL2

While the data reported so far provide multiple therapeutic options for patients with r/r CLL, disease progression and especially refractory disease after pretreatment with both inhibitors of BTK and BCL2 still pose a challenge in daily clinical practice. CLL in double-refractory patients is often enriched for high-risk disease characteristics and OS is heavily reduced with a reported median OS of less than 8 months for patients without Richter’s transformation [40]. Approved therapeutic options are very limited and only able to achieve short-lasting disease control (if any): Treatment with PI3K-inhibitors such as IR was associated with an ORR of only 46% and a PFS of 5 months, while treatment with BTKI in patients refractory to a previous BTKI achieved similar numbers with an ORR of 50% and a PFS of 4 months [38]. Combined treatment approaches such as venetoclax plus ibrutinib appear to hold some promise, as Hampel et al. [41] were able to report an ORR of 100% and a median OS of 27 months in 11 patients with double-refractory CLL.

Due to these sobering data exposing an urgent medical need we strongly recommend enrolling patients with double-refractory CLL to well-designed clinical trials exploring novel therapeutic options.

**Fig. 1:**
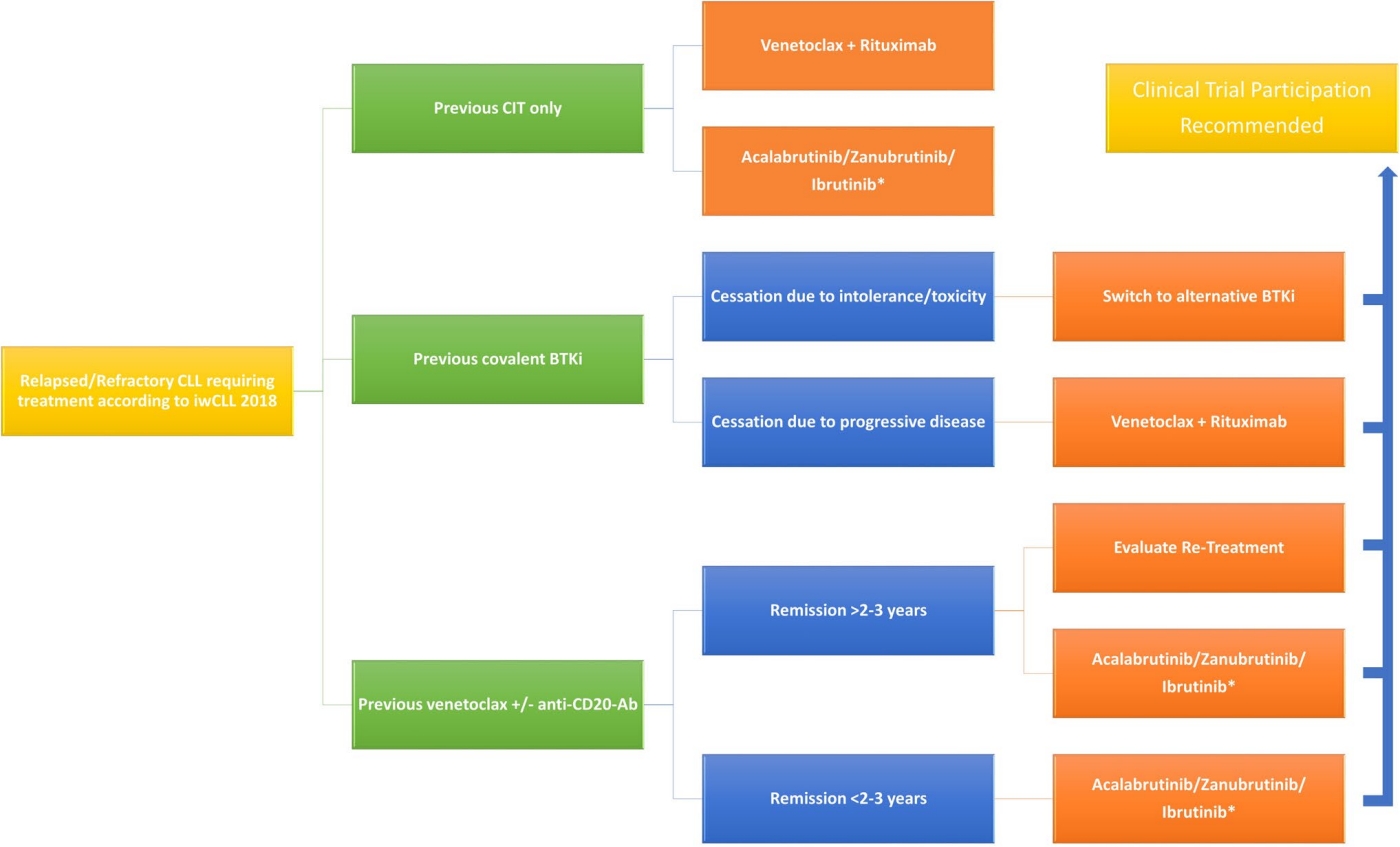
Proposed treatment algorithm for relapsed/refractory CLL. CLL: chronic lymphocytic leukemia. CIT: chemoimmunotherapy. BTKI: covalent BTK inhibitor. Ab: antibody. ^*^Second-generation BTKi (acalabrutinib, zanubrutinib) should be preferred over ibrutinib due to their side-effect profile

Figure 1 shows our current therapeutic algorithm for patients with r/r CLL who have received at least one prior treatment.

## Emerging Therapeutic Options in Relapsed/Refractory CLL

### Noncovalent BTK-inhibition

Despite the high ORR and mostly durable remissions achieved with currently available covalent first- and second-generation BTKI in r/r CLL, long-term follow-up data of prospective clinical trials document that > 50% of patients discontinue ibrutinib within 5 years on treatment [8, 9, 14, 25]. Besides toxicity in approximately one quarter of patients, disease progression has been the second most common limiting event in about 20% of patients [42]. Indeed, mutations at the BTKI binding residue C481 (found in about 56% of patients), gain-of-function mutations of PCLG2 (8% of patients), or both (16% of patients) conferring resistance are frequently encountered [28–30].

Thus, there is a remaining need to overcome these mechanisms of resistance associated with covalent BTKI with novel therapeutic approaches.

Noncovalent binding reversible BTKIs were developed that do not engage on the C481 binding site and may potentially inhibit both C481-mutant and unmutated BTK with similar efficacy.

Pirtobrutinib is a highly selective noncovalent reversible BTKI with the currently most advanced clinical data in r/r CLL. Pharmacokinetic data suggests a favorable oral bioavailability and longer half-life of approximately 19 h. Through its reversible binding mode, pirtobrutinib facilitates stable target coverage independent on intrinsic BTK turnover [43]. Within the phase 1–2 BRUIN basket study, 247 CLL patients previously treated with a covalent BTKI and a median of prior therapies of 3-40.5% had also been exposed to a BCL2 inhibitor, 18.2% to a PI3K inhibitor – were treated with pirtobrutinib at a standard dose of 200 mg once daily until disease progression or unacceptable toxicity. 76.9% of patients had discontinued prior BTKI due to disease progression, 23.1% due to toxicity. 37.8% of evaluable patients (84/222) showed a BTK C481 mutation, 8.1% patients (18/222) had a PLCG2 gain-of-function mutation at study entry. ORR was 82.2% for all 247 patients and 79.0 % for double-exposed patients (n=100, inhibitors of both BTK and BCL2) when PR with lymphocytosis was included. With a median follow-up of 19.4 months, median PFS was 19.6 months for the entire cohort, 16.8 months for double-exposed patients, and 13.8 months for those who had been treated with CIT, BTKI and inhibitors of BCL2 and PI3K. ORR and PFS estimates appeared independent from BTK C481 mutation status and 56% of patients with a PLCG2 mutation achieved a response. Eighteen-month OS was 80.5% for the entire cohort at a median follow-up of 22.6 months [44•].

The ≥3 grade toxicity profile associated with covalent BTKI was low, with atrial fibrillation in 1.3%, hypertension in 3.5%, and hemorrhage in 2.2% of patients respectively. Most common ≥3 grade AEs were infections (28.1%) and neutropenia (26.8%). No ventricular arrhythmias or sudden cardiac deaths were observed.

Dose reductions due to treatment-related AEs were required in 4.5% of patients, permanent treatment discontinuations in 2.6% of patients. Given the encouraging efficacy and feasible safety profile in these high-risk CLL patients with unmet medical need, several randomized clinical trials have been launched to better character the position of pirtobrutinib within the treatment landscape of CLL: BRUIN-CLL-313 (NCT05023980, pirtobrutinib vesus BR in untreated CLL), BRUIN-CLL-314 (NCT05254743, pirtobrutinib versus ibrutinib in untreated or r/r CLL), BRUIN-CLL-321 (NCT04666038, pirtobrutinib versus investigator’s choice (IR or BR) in r/r CLL) and BRUIN-CLL-322 (NCT04965493, pirtobrutinib/venetoclax versus VenR in r/r CLL).

Nemtabrutinib is another noncovalent reversible BTKI in early clinical development in the phase 2 BELLWAVE-001 dose-expansion study. At ASH 2022, an update on efficacy and safety among 57 patients with r/r CLL (≥ 2 prior therapies, including patients with a BTK C481 mutation or intolerant to covalent BTKI) treated with nemtabrutinib at a target dose of 65 mg was presented. With a relatively short follow up of 8.1 months ORR was 56% and estimated median duration of response was 24.4 months. The most common AEs included dysgeusia (21%), neutropenia (20%), fatigue (13%), nausea and thrombocytopenia (12% each) as well as diarrhea and hypertension (10% each). Most frequent ≥3 grade AEs were neutropenia (17%) and thrombocytopenia (5%). AEs led to treatment discontinuation in 13% of patients with no deaths being documented as treatment-related [45]. As such, at this early stage of development, treatment-related drug discontinuations appear to be higher with nemtabrutinib (13% versus 2.6%), possibly as a result of inferior kinase selectivity compared to pirtobrutinib. Longer follow-up and increased patient numbers are needed to better define the role of nemtabrutinib next to pirtobrutinib.

### CAR-T-Cell-Therapy

Chimeric antigen receptor T (CAR-T)—Cells have revolutionized treatment in B-cell lymphomas as well as acute lymphoblastic leukemia and initial results in CLL from more than 10 years ago have shown remarkable efficacy [46, 47]. A prospective phase I/II-trial involving 19 patients treated with a CD19-CAR-T previously treated with BTKI showed promising results with an ORR of 83% and a CR rate of 22%. After a median follow-up of 12 months, the 1-year PFS and OS-rate in all included patients were 38% and 64%, respectively [48].

The largest dataset to date stems from the prospective TRANSCEND CLL 004 phase I trial which tested lisocabtagene-maraleucel, a CD19-CAR, in 137 patients with r/r CLL and at least two lines of previous therapy, including a BTKI. The primary efficacy analysis set consisted of 70 patients which were also pre-treated with venetoclax and exhibited high-risk disease characteristics. The ORR and CR rate in this group was 43% and 18%, respectively, with an impressive rate of undetectable MRD of 63% in peripheral blood and 59% in the bone marrow [49]. The median PFS and OS for responders was not reached, paving the way for further studies into this promising therapy.

### Bispecific Antibodies/T Cell Engangers (BITe)

Although presenting impressive results, logistical and socioeconomic challenges remain with the use of CAR-T therapy, so far limiting their use to specific settings. Aiming to fill that gap or rather add to the increasing arsenal, bispecific antibodies are promising therapies using the innate immune system by linking therapeutic targets such as CD20 with CD3-positive T cells. Epcoritamab has demonstrated a tolerable side-effect profile in CLL [50] and together with mosunetuzumab's impressive efficacy results in other indolent and aggressive B non-Hodgkin lymphomas [51] the results of ongoing trials (NCT05091424, NCT04623541) in CLL are therefore eagerly awaited.

### Allogenic Transplant

Considering the aforementioned advances in therapy, allogeneic stem-cell transplant (SCT) has an increasingly subordinate role in CLL but remains the, so far, only therapeutic option offering a hope of cure. Due to the ever-improving PFS and OS rates and the median age at first diagnosis of around 70 there is little data on the use of allogenic SCT in the setting of novel therapies.

Kim et al. [52] retrospectively analyzed 108 patients receiving allogenic SCT at their institution, of which 30 were previously treated with novel agents. After a median follow-up of 36 months the median OS and PFS were not reached. The 3-year cumulative incidence of treatment-related mortality and progression was 7% (95% CI 1–19%) and 21% (95% CI, 8–38%), respectively. Similar data was reported from a multicenter retrospective cohort study involving 65 patients, of which 16 were deemed double refractory: After a median follow-up of 27 months the median OS and PFS were not reached [53]. These promising results support the potential of allogenic SCT, even in the setting of novel agents, however, median ages of 60 and 50 and HCT-CI-scores of 0 for 38 and 43% of patients, respectively, underline the need for careful patient-selection.

## Conclusion

CLL treatment has truly been revolutionized in the last decade with various novel agents, foremost inhibitors of BTK and BCL2, entering the clinical stage. Outperforming CIT in both efficacy—particularly in genetic high-risk constellations—and tolerability chemotherapy-free regimens have become treatments of choice in first and subsequent lines of therapy. Albeit, even with novel therapies the adverse prognostic impact of TP53-alterations, unmutated IGHV status and karyotype complexity still remains to be fully compensated, predisposing most CLL patients to multiple lines of therapy in the long run. As such, acquired resistance and/or drug-associated toxicity remain frequently encountered issues to overcome in daily clinical practise. Herein, second-generation, more selective BTKIs have become an important treatment alternative at relapse for CLL patients formerly discontinuing ibrutinib due to AEs or those with cardiovascular comorbidities. In CLL patients with disease progression on continuous treatment with novel drugs, a class-switch is generally recommended as acquired resistance is commonly observed. In case of disease progression after fixed-duration treatment with venetoclax-based combinations, re-treatment may be a viable and elegant option to consider in patients with late relapse (> 2 years) in the absence of acquired resistance mutations. Treatment options for CLL patients with double-refractory disease after exposure to both inhibitors of BTK and BCL2 remain an unmet medical need with currently approved agents usually facilitating only short-lived remissions and patients are strongly encouraged to engage in prospective clinical trials investigating novel drugs. Encouragingly, novel approaches such as noncovalent BTKI, bispecific antibodies or CAR-T therapy have shown remarkable activity in this difficult-to-treat subgroup. By tailoring treatment sequences based on the individual CLL cells’ biology, patients’ comorbidities and preferences, we can move closer to achieving personalized and steadily more effective management of CLL.
